# Increasing the rigor of body composition and adiposity measurement in multiple sclerosis research

**DOI:** 10.3389/fneur.2026.1737057

**Published:** 2026-02-25

**Authors:** Meghan G. Taylor, Amy Goss, Brooks C. Wingo

**Affiliations:** 1 Department of Nutrition Sciences, University of Alabama at Birmingham, Birmingham, AL, United States; 2 Department of Occupational Therapy, University of Alabama at Birmingham, Birmingham, AL, United States

**Keywords:** adipose tissue, body composition, dual energy X-ray absorptiometry, magnetic resonance imaging, multiple sclerosis

## Abstract

Multiple sclerosis (MS) is an immune-mediated neurodegenerative disease that affects nearly 1 million adults in the United States, and over half of this population also has overweight or obesity. The compounding effect of multiple disease states could increase disease progression and worsen MS symptoms. MS researchers frequently use anthropometric measures, such as BMI and waist circumference, as an assessment of obesity. However, these measurements do not provide a direct assessment of types or location of adipose tissue, which may provide a more accurate assessment of adiposity-related health risk. The main objectives of this mini review are to provide a brief overview of current adiposity measurement techniques in MS research and highlight potential benefits of using more rigorous indirect and direct techniques to measure total, regional, and specific fat depots.

## 1 Introduction

Multiple sclerosis (MS) is a progressive neurodegenerative autoimmune disease that affects nearly 1 million adults in the United States (1). The demyelination, inflammation, and neuronal loss of the condition leave adults with MS to face a myriad of physical and cognitive symptoms, including neuropathic and musculoskeletal pain, muscle weakness, fatigue, and depression (2, 3). Researchers have also found that approximately half of this population also has overweight or obesity (4), and this may speed MS-related disability progression.

Obesity may affect MS disease progression in two ways. First, it directly contributes to low-grade systemic inflammation and neuroinflammation through the release of pro-inflammatory cytokines and adipokines including TNF-α, IL-6, and leptin (5–8). This inflammation is associated with cognitive dysfunction and physical disability (9–11). Obesity also indirectly impacts MS by increasing the risks of other cardiometabolic conditions including hypertension and type 2 diabetes, which are also inflammatory and are associated with MS progression and disability.

Higher fat mass has been linked to lower brain volume in otherwise healthy adults at midlife (4). In adults with neurodegenerative diseases such as MS, Alzheimer’s disease (AD), and Parkinson’s disease (PD), increased release of pro-inflammatory cytokines and insulin resistance originating from excess adiposity have been correlated with an increased rate of disease progression and hospitalization (10, 11). Rather than a simple measure of total fat mass, specific depots appear to drive the deleterious influence of excess adiposity on health, with visceral and ectopic adipose deposition being particularly harmful for brain health.

Visceral adipose tissue (VAT) has been correlated with lower brain volume in adults at midlife, which puts those with neurodegenerative diseases at an increased risk of disease progression (12). One systematic review focused on associations between regional adiposity, cognitive function, and dementia-related brain changes showed links between abdominal fat depots and brain health (9). Results from 33 clinical trials based on adults from the general population showed that higher VAT and hepatic fat were associated with cognitive decline; however, to our knowledge, no research has been published on measuring VAT directly in adults with MS. This suggests that greater emphasis on measuring specific adipose tissue depots may need to be considered in research focused on disease management in adults with MS, who are already at greater risk of cognitive decline.

Despite a growing recognition of the impacts of excess adiposity on neurodegeneration, most research in MS does not include the rigorous measures of body composition needed to fully explore the mechanisms through which the amount and location of adipose tissue may lead to increased disease activity. The objectives of this mini review are to briefly discuss the measures of adiposity frequently used in current MS research, highlight key benefits and limitations of these methodologies, and present the benefits of combining indirect and direct techniques.

## 2 Limitations of current research measures

Anthropometric measures, such as body mass index (BMI), waist circumference, waist to height ratio (WHtR) and waist to hip ratio (WHR), are frequently used as measures of obesity and cardiovascular disease risk in current MS research. These can be helpful estimates of obesity when used in combination. For example, BMI and waist circumference have both been inversely associated with cognitive performance (11). BMI is the most widely used clinical assessment for obesity at a population level and classifies an individual as underweight (BMI < 18.5), normal weight (BMI = 18.5–24.9), overweight (BMI = 25.0–29.9), or one of three classes of obesity (BMI ≥ 30.0) (11). BMI relies on a weight to height ratio (weight (kg)/height(m)^2^) to estimate excess adiposity but lacks the ability to determine differences between fat mass and lean mass or account for fat distribution (11, 13, 14). Research has shown that adults with MS have more fat mass when compared to BMI-matched healthy controls. According to one cross-sectional study comparing BMI to dual-energy X-ray absorptiometry (DXA) measures of lean and fat mass in adults with MS and non-MS controls, BMI was associated with an underestimation of adiposity and overestimation of lean mass in adults with MS (15). Recent reviews of clinical research in MS suggest that fat distribution is linked with disability, but many studies limit measures of adiposity to BMI. Few clinical studies followed up with more extensive body composition techniques to distinguish between specific adipose tissue depots (16, 17).

Waist circumference, waist to height ratio, and waist to hip ratio are also used in clinical practice and research to assess cardiometabolic health risk. Similar to BMI, waist circumference and waist-to-hip ratio cannot distinguish between fat and lean mass but have the advantage of being directly associated with central obesity. For example, using waist circumference as a risk assessment for VAT has been well-documented in healthy populations, and researchers have highlighted it as a more effective tool for determining adipose-related disease risk than BMI alone (18). A recent systemic review and meta-analysis conducted by Giannopapas et al. (19) addressed the clinical use of waist circumference in combination with BMI to predict disability progression in MS. Outcomes from their research indicate that using these measures in combination creates a stronger predictor of cardiovascular risk in adults with MS than using either in isolation (19). As this research highlights, combining these two measurement tools could provide a more holistic estimation of how adiposity may be distributed to the visceral depot and lead to worsening health outcomes.

Using anthropometric measures may be a practical proxy measure for determining obesity, but incorporating body composition analyses is needed to understand the mechanisms through which excess adiposity impacts MS. Bioelectrical impedance (BIA) has been used in clinical research as an accessible technique for measuring fat mass in various populations with good correlation between it and DXA, but little research has addressed BIA in relation to nutritional status in adults with MS (20, 21). BIA provides a measurement of fat mass and fat free mass using low-volt electrical currents with monitored levels of impedance (22). Higher impedance reflects higher fat mass, and lower resistance suggests greater fat-free mass. However, this tool is heavily influenced by hydration status and could provide inaccurate measures due to factors such as age and water intake. While multi-frequency BIA devices offer more reliability than traditional, single-frequency BIA devices (23), hydration status may still skew interpretations of lean mass and fat mass in the general population (24–26). Research in MS has similar findings. For example, a recent study using BIA for fat mass evaluation in adults with MS noted that overestimations of fat free mas and underestimations of fat mass could occur due to changes in hydration status accompanying glucocorticoid therapy, a common treatment in MS (27). These medications cause water and sodium retention, which contributes to increased soft tissue hydration and potentially leads to misinterpretations of fat free mass and fat mass with BIA. Although typically non-invasive, cost-effective, and portable, this indirect technique may lack precision and accuracy due to measurement error and the inability to obtain tissue-specific measurements (15, 26). Studies in MS have used central adiposity estimates from BIA measures, but the application of BIA in evaluating VAT should be used with caution, considering BIA’s variable outcomes when compared against MRI and CT (26). In MS, there have been no known studies validating BIA algorithms of VAT estimation against a direct measure such as MRI or CT in populations with MS (Table 1).

**Table 1:** Characteristics of adiposity measurement techniques.

Measurement technique	Total fat mass	Regional fat mass	Intermuscular adipose tissues (IMAT)	Visceral adipose tissue (VAT)	Strengths	Limitations
Body mass index (BMI)					Inexpensive Fast Feasible for large population studies No use of ionizing radiation or strong magnet Safe for all populations	Incapable of measuring regional adiposity or differentiating from fat and lean mass Inaccurate for certain populations (Over- and underestimation of fat mass)
Waist circumference		(abdominal estimate) ✓			Inexpensive Fast Feasible for large population studies Improved estimate of abdominal adiposity when used in combination with other anthropometric measures (e.g., weight/height) No use of ionizing radiation or strong magnet Safe for all populations	Indirect measure of adiposity Incapable of estimating total fat mass
Bioelectrical impedance (BIA)	(estimate) ✓				Feasible for large population studies Fast No use of ionizing radiation or strong magnet	Indirect measures of fat mass and lean mass Accuracy depends on hydration status, age, and body size Not safe for all populations
Dual-energy X-ray absorptiometry (DXA)	✓	✓		(estimate) ✓	Minimal use of ionizing radiation Able to provide total body and regional measures of adiposity Less expensive compared to MRI and CT	Indirect measures only for regional adiposity Not safe for all populations
Magnetic resonance imaging (MRI)			✓	✓	No use of ionizing radiation High accuracy for adiposity measurement Direct measures of adipose tissue volume	Participants must remain completely still for image clarity High cost may not be feasible Metals from implanted devices are not allowed Size of the bore limits use in higher BMIs
Computed tomography (CT)			✓	✓	High accuracy for adiposity measures Direct measure of adiposity	Use of ionizing radiation Full body measures are not feasible High cost may not be feasible Not safe for all populations

## 3 Benefits of more rigorous adiposity measures

Differentiating between subcutaneous, visceral, and intermuscular fat is necessary to understand how obesity affects MS disease progression. DXA, MRI, and computed tomography (CT) have been validated as the most reliable techniques for measuring adiposity in adult populations, but DXA and MRI have proven to be particularly helpful for adults with MS as these techniques detect specific locations of adiposity over time without the risk of high levels ionizing radiation (28). DXA estimates regional fat distribution, and MRI directly measures specific adipose tissue depots. Additionally, MRI can differentiate between subcutaneous, visceral, and intermuscular adipose tissues. This is a key strength of MRI and CT since DXA does not have this capability yet.

### 3.1 DXA

Originally created to measure bone density, DXA uses ionizing radiation to measure fat and lean soft tissue through mass attenuation (29). Two energy levels are produced from filtered X-ray beams, and mass attenuation coefficients are measured through an energy detector (29). Mass attenuation depends on tissue thickness, X-ray intensity, radiation type, and material density and composition, and scan results are reported using 2-D pixel images (29).

DXA can also be used to estimate regional body composition including fat and lean tissue mass in the legs, trunk, gynoid and android regions, and ratios of waist-to-hip mass and trunk-to-limb mass may then be calculated, allowing for better estimation of central adiposity (30, 31). Importantly, DXA can detect changes in body composition over time, even in the absence of body weight change, as demonstrated in diet and exercise interventions in which weight and BMI did not change, but fat mass significantly decreased while lean mass increased. For example, DXA has been used in randomized controlled trials investigating the impact of aerobic and strength-training on body composition in people with overweight or obesity and at risk for cardiometabolic disease and prediabetes. Results indicated that participants had increased lean mass and reduced fat mass without significant changes in total body mass (32, 33). This highlights the beneficial effects of lifestyle interventions beyond weight loss that cannot be detected with less rigorous anthropometric measures.

Another advantage of DXA is its capacity to accommodate adults in higher classes of obesity who may not be able to be measured with techniques with size limitations. DXA provides a measure of whole-body soft tissue composition, including fat mass and body fat percentage, and can provide estimations of composition for those in higher BMI classes of obesity (34). Considering the percentage of adults with MS or other neurodegenerative diseases that also have obesity, a tool with this capability offers valuable insight into a targeted range of people in this population. If researchers’ goal is related to total fat mass and lean tissue estimates, then DXA should be considered as a supplementary measure to BMI or a comparison measure for more sensitive imaging techniques in adults with MS and obesity.

While DXA is highly regarded and beneficial for measuring adiposity in adults with MS, its use has documented limitations both in the general population and in adults with MS. One of DXA’s main limitations is its use of ionizing radiation, which is of concern for high-risk populations, such as women who are pregnant (29). The radiation exposure is lesser when compared to techniques such as CT but should still be evaluated if appropriate and necessary. Additionally, DXA, while able to measure whole body or regional fat mass and lean mass, can only produce 2-D pixel images and is limited to indirect estimates of specific adipose tissue types, such as VAT. In a recent review, researchers showed that a limitation of DXA is the potential inaccuracy of scans in adults with MS and obesity due to variability of lean mass and fat mass (16). This is particularly important for the interpretation of MS research that uses DXA to report VAT, as lean and fat mass are used to estimate VAT. Another limitation of DXA is that its lean mass estimates are affected by hydration status, and loss of water could be interpreted as loss of lean mass (35). This further highlights the need for ongoing investigations of fat distribution in adults with MS using techniques with the ability to directly measure fat depots.

### 3.2 MRI

MRI uses a strong magnetic field and pulsed radiofrequency (RF) to measure naturally abundant hydrogen protons. Lean tissue and adipose tissue composition differences affect precessional proton frequency and relaxation responses to the magnetic field after pulses (36). MRI sequences are based on targeted tissue measures, and results are reported as 3-D images. MRI does not require ionizing radiation. Researchers may use either DXA or MRI to conduct regional analyses, but MRI provides an added advantage of direct measurement of specific adipose tissue depot volume. For example, in a study investigating the relationship between low-fat and low-carbohydrate diet interventions, DXA indicated improvements in total fat mass, which was further detailed by MRI to be reductions specifically in visceral adiposity (37). MRI can also measure intermuscular adipose tissue, as well as organ lipid and volume (36). Given the prevalence of metabolic conditions among people with MS, and the correlation between obesity, type 2 diabetes, and poor MS outcomes, MRI techniques could prove valuable in understanding the mechanisms underlying these associations.

While MRI is ideal for identifying visceral, subcutaneous, and intermuscular fat in adults with MS, it does have limitations. Although MRI provides detailed analysis of specific adiposity stores, it is not recommended for full body measurement. Because imaging is conducted as slices, the imaging would require a long time in the scanner, and the analysis of a full body measurement would be difficult. While recent MRI technology offers rapid whole-body scans, the cost is higher than current standard imaging and may not be feasible. Therefore, it is recommended that researchers use DXA for mass estimates and MRI for directly measuring tissue volume.

Another significant limitation is cost. Depending on a researcher’s budget, MRI may not be a feasible option. Logistics of the machine size, sound, setup, and research participants’ willingness and ability to adhere to MRI protocols could also be a deterrent. For example, if an individual has claustrophobia or an implanted metal device, such as a pacemaker, MRI may not be appropriate for their emotional well-being or safety. Moreover, adults with MS typically complete MRI scans for initial diagnosis, monitoring brain lesions over time, and overall disease progression. Undergoing additional MRIs for participation in a research study could result in increased participant burden and prevent some individuals from agreeing to participate.

## 4 Discussion

The role of obesity in brain health and MS progression is increasingly acknowledged, and more MS research now includes measures of body weight and estimates of obesity. Research shows a relationship between increased BMI and reduced physical mobility and cognitive function over time, with one case-controlled study reporting that steadily increasing BMI over a 15-year time-span was associated consistently with increased EDSS scores, reflecting reduced physical mobility, and a decline in symbol digit modality test (SDMT) scores, a commonly used measure of cognitive function in adults with MS (38). While these findings are important, research in the general population and in related neurodegenerative conditions demonstrates the need to shift focus from simple measures of body weight to the specific measures of location and tissue-specific adiposity (10).

Current MS research primarily uses BMI and body weight to measure obesity, which can further the understanding of the associations between excess adiposity and MS, but to truly understand mechanisms through which obesity leads to MS progression, it is necessary to use more specific measures of adiposity. Excess adiposity is known to contribute to the release of pro-inflammatory cytokines, worsened insulin sensitivity, and an increased risk of ectopic fat deposition, such as in the muscle, liver, and pancreas (39). Consequently, these may contribute to metabolic dysfunction, hormonal imbalances, disruption of the blood–brain barrier, and reduced brain health (40). More specifically, VAT has been linked to reduced brain health and a potentially heightened risk of developing AD (41). VAT contributes to increased circulating pro-inflammatory cytokines, such as TNF-a and IL-6, which can pass through the blood–brain barrier and play a role in neuroinflammation and increased oxidative stress. VAT has also been shown to contribute to insulin resistance, highlighting both the direct and indirect impacts of obesity on MS. Glucose is the brain’s primary energy source, and impairment in its uptake has been associated with a reduction in brain function and faster decline (42). These effects from VAT compounded with active symptoms of MS could place this population at higher risk for neurodegeneration and disease progression, but to date, most MS trials have not collected the types of measures necessary to conduct these analyses in adults with MS. While recent studies have reported associations with central adiposity and MS symptoms including pain, fatigue, and depression, this research used indirect methods to estimate VAT (e.g., waist circumference) (43). There have been no published reports of using MRI or CT to directly measure VAT in adults with MS. Additionally, while most existing data is cross sectional in nature, longitudinal studies that examine change in direct measures of VAT overtime are also needed.

Specific measures of obesity including DXA and MRI are also necessary to understand changes in body composition independent of weight loss. Lifestyle interventions including physical activity and diet interventions have demonstrated improved MS outcomes including fatigue and depression (34–36). While some of the benefits of these interventions may be due to weight loss, they also lead to preferential changes in body composition. For example, a 12-week exercise intervention in adults with excess adiposity showed that participants improved body composition after following high-intensity interval training (HIIT), resistance training, and combined training programs even without weight loss (44). When these changes in body composition occur without weight loss, they cannot be detected by BMI or body weight alone. Moreover, the metabolic and neurological impacts of these interventions cannot be fully understood unless studied within the context of body composition, rather than a simple obesity categorization.

It is important to acknowledge that adiposity is only one aspect of body composition. Lean mass is also important in the context of MS, since this population is at risk for muscle atrophy and dysfunction (45). In adults with MS and related neurodegenerative conditions, combined aerobic and anaerobic activity has shown to improve the quality of muscle tissue by increasing muscle mass and improving muscle quality through reductions in intermuscular adipose tissue (IMAT) (46, 47). Increasing muscle mass contributes to better overall physical function in adults with MS by improving walking, mobility, and strength (48, 49). Excess adiposity creates greater risk for fat infiltration into muscle tissue, such as IMAT, which is associated with functional decline in MS (16). Using IMAT as a measure of muscle health in MS research regularly could provide a better understanding of physical function over time. Using more advanced techniques that integrate measures of muscle mass and muscle health with adiposity could provide a more thorough view of participant health and risk of disease progression.

In conclusion, to fully understand the role of obesity in MS progression, multiple body composition measures should be incorporated into MS research. Anthropometric measurements, such as BMI and waist ratios, are cost-effective, easily implemented estimates of obesity but are not precise measures of adiposity. BIA has been used for distinguishing fat mass from fat free mass in MS studies with inconsistent outcomes. MRI, CT, and DXA are the best ways to measure adiposity and have been validated in this population, but they are not frequently used, due, in part, to the high cost and participant burden. Moving forward, researchers should consider adding multiple measures of adiposity to study protocols. For example, body mass may be used as a general descriptor of weight or weight change among study participants, while DXA can be added to estimate total and regional body fat, and MRI scans can be used to provide further distinction between specific adipose tissue depots.
